# TCN-5mC: a predictor of 5-methylcytosine sites based on multi-feature fusion and TCN-inspired block networks

**DOI:** 10.3389/fgene.2026.1739720

**Published:** 2026-02-03

**Authors:** Cunwen Liu, Xuan Xiao, LongChang Wan, WeiZhong Lin

**Affiliations:** 1 School of Information Engineering, Jingdezhen Ceramic University, Jingdezhen, China; 2 School of Information Engineering, Jiangxi Art & Ceramics Technology Institute, Jingdezhen, China; 3 School of Information Engineering, Jingdezhen University, Jingdezhen, China

**Keywords:** 5-methylcytosine site, CBAM, deep learning, promoter, TCN

## Abstract

Accurate identification of 5-methylcytosine (5 mC) sites in promoter regions is crucial for understanding epigenetic regulation, but experimental methods remain costly and time-consuming, highlighting the need for reliable computational prediction tools. While existing deep learning approaches, such as BiLSTM-based, Transformer-based, and pretrained language models, have advanced the field, opportunities remain for further improvements in capturing long-range dependencies and handling imbalanced genomic data. Here, we present TCN-5mC, a deep learning model that integrates Temporal Convolutional Networks (TCN) inspired block with Bidirectional Gated Recurrent Units (BiGRU) and employs hybrid One-Hot and Nucleotide Chemical Property feature encoding. This architecture is designed to more effectively model both extended sequence contexts and local patterns. The model achieves high predictive performance on imbalanced datasets from lung cancer cell lines, with AUC values of 0.967 and 0.989 on two independent test sets, outperforming existing methods in specificity, accuracy, MCC, and AUC. The model thus provides a robust, high-throughput computational tool for 5 mC site prediction, with promising potential for epigenetic research and biomarker discovery.

## 1 Introduction

Aberrant DNA methylation is an established driver of pathogenesis, mechanistically linked to a broad spectrum of diseases. These include various carcinomas (e.g., liver, lung, kidney, cervical, ovarian, breast) (Chen et al., 2016; Devi et al., 2021; Tucker et al., 2018; Vanaja et al., 2009; Zhang et al., 2021), Alzheimer’s disease (Shinagawa et al., 2016), Parkinson’s disease (Su et al., 2015), and diabetes-related obesity (Zhang et al., 2015; Lecorguillé et al., 2023). Among various epigenetic modifications, 5-methylcytosine (5 mC) is the predominant and functionally vital form of DNA methylation. It plays a central role in regulating gene expression, maintaining genome stability, and influencing biological development and disease progression (Jones, 2012). 5mC is involved in processes such as gene silencing, X-chromosome inactivation, and genomic imprinting (Moore et al., 2013; Wang et al., 2016). Consequently, 5 mC dysregulation is a focal point in epigenetic research, with implications extending beyond cancer to autoimmune rheumatic diseases like rheumatoid arthritis and systemic lupus erythematosus (Rodríguez-Ubreva et al., 2019). These findings underscore the significance of accurately identifying 5 mC sites, which could provide valuable insights into disease mechanisms and potential therapeutic targets (Nassiri et al., 2020).

However, conventional experimental methods for 5 mC detection, such as bisulfite sequencing (Li and Tollefsbol, 2011), oxidized bisulfite sequencing (Booth et al., 2013), and Aza-IP (Khoddami and Cairns, 2014), are costly and time-consuming for large-scale or clinical applications, which presents a significant technical bottleneck. Consequently, developing accurate and efficient computational tools to predict 5 mC sites is essential. High-confidence prediction of 5 mC loci, especially in regulatory regions, holds the promise not only of deepening our biological understanding but also of enabling the discovery of accessible epigenetic biomarkers for diagnostic applications.

Following earlier machine learning approaches, such as support vector machines (Chen L. et al., 2022), XGBoost classifier (Liu et al., 2022), and stacking strategies (Chai et al., 2021), which often faced limitations in scalability and complex feature engineering, deep learning techniques have rapidly become dominant for 5 mC site prediction, driving significant leaps in accuracy. The evolution of deep learning models for this task showcases a progressive integration of sophisticated architectures.

Initial efforts, such as iPromoter-5mC (Zhang et al., 2020), demonstrated the viability of using simple Deep Neural Networks (DNNs) with basic encoding schemes. To better model the sequential nature of DNA, subsequent work introduced Bidirectional Long Short-Term Memory networks (BiLSTM) to capture long-range dependencies in DNA sequences (Cheng et al., 2021). The cross-species validation framework in m5c-iDeep further revealed RNN architecture capabilities in distinguishing species-specific methylation patterns (Malebary et al., 2024). Feadm5C (Bilal et al., 2025) was proposed, which was a novel method that integrates molecular graph features (MGF) with BiLSTM to enhance the prediction of RNA 5-methylcytosine (m5C) sites. This combined approach demonstrated superior performance and achieved robustness across multiple species.

Further innovation emerged with hybrid architectures. For example, DGA-5mC (Jia et al., 2023) fused a modified Densely Connected Convolutional Network (DenseNet) for local feature extraction with a Bidirectional Gated Recurrent Unit (BiGRU) to capture contextual information across longer ranges. And the model employed diverse encoding schemes (One-Hot, Nucleotide Chemical Property, Nucleotide Density) to enrich feature representation.

More recently, Transformer-based models have been applied to distill global sequence features. Fu et al. (2025) proposed an M5C site prediction model based on the Transformer architecture, which automatically constructs DNA sequence features through positional and 6-mer embeddings, eliminating the need for complex feature engineering. Similarly, Kinnear et al. (2025) introduced the Deep5mC model, which also employs a deep learning Transformer framework to predict 5 mC methylation status in DNA sequences. Deep5mC integrates token embedding, positional embedding, and CNN to capture local and long-range dependencies within DNA sequences, significantly enhancing the accuracy of 5 mC predictions.

The most recent breakthrough leverages transformer-based architectures pretrained on large genomic datasets, exemplified by BERT-5mC (Wang et al., 2023). By fine-tuning the DNABERT model, a multi-scale DNA language model pretrained on massive genomic datasets, BERT-5mC eliminates the need for manual feature engineering. The transformer’s self-attention mechanism enables the direct learning of complex contextual dependencies from raw sequences, achieving state-of-the-art predictive performance.

Concurrently, ensemble learning strategies have been employed to enhance prediction accuracy and reliability. For instance, m5c-Seq (Abbas et al., 2024) designed a hierarchical fusion framework that aggregated probabilistic outputs from XGBoost, SVM, and CatBoost submodels into a Logistic Regression (LR) meta-classifier, improving the model’s predictive performance. Similarly, MLm5C (Kurata et al., 2024) optimized sequence window length and employed sequential forward search to select 20 base models from 44 candidates for feature stacking, thereby significantly enhancing the precision of human RNA prediction. Furthermore, m5C-iEnsem (Bilal et al., 2025)utilized ensemble learning techniques, including Bagging and Boosting, to process encoded data and predict 5 mC sites with superior accuracy and generalization. These studies collectively highlight the efficacy of ensemble learning in advancing the field of RNA modification prediction, demonstrating the potential for further innovation and application in biological and medical research.

Alongside these developments, the temporal convolutional network (TCN) has emerged as a powerful alternative for sequence analysis. TCNs offer distinct advantages over traditional recurrent models, including flexible receptive field control through dilated convolutions and more efficient capture of long-range dependencies without a significant increase in computational cost. Their efficacy has been demonstrated in various bioinformatics tasks, such as neuropeptide prediction (Chen S. et al., 2022) and promoter identification (Raza et al., 2023), highlighting their potential for genomic sequence analysis.

In this study, we propose a novel deep learning framework, TCN-5mC, to predict 5 mC sites. Our model utilizes a hybrid feature encoding approach (One-Hot and NCP) and integrates a TCN-inspired block with a BiGRU. This architecture leverages the TCN’s strength in capturing long-term dependencies and the BiGRU’s ability to model bidirectional sequential contexts. We evaluated TCN-5mC using 5-fold cross-validation and independent testing on two benchmark datasets. The results demonstrate that our model consistently outperforms existing state-of-the-art methods, confirming the effectiveness of the TCN-inspired block-BiGRU hybrid approach for 5 mC site prediction.

## 2 Materials and methods

### 2.1 Benchmark datasets

A high-quality benchmark dataset is essential for accurate model construction. In this study, we used a dataset created by Xiao et al. from the Cancer Cell Line Encyclopedia (CCLE) (Barretina et al., 2012), which contains promoter 5 mC site information. Given the high incidence and mortality rates of lung cancer, this study focused on human small cell lung cancer (SCLC) and non-small cell lung cancer (NSCLC) to analyze the distribution of 5 mC sites in promoters. Through extensive validation, 41 base pairs (bp) were determined to be the optimal sequence length for promoter 5 mC site prediction. A cytosine (C) base was labeled as a true 5 mC modification site if its methylation level was greater than zero; otherwise, it was classified as a pseudo 5 mC site. To ensure the uniqueness of each sequence, the CD-HIT tool (Fu et al., 2012) was used to remove sequences with over 80% similarity. This process resulted in two benchmark datasets. Benchmark dataset 1, which focuses on small cell lung cancer (SCLC), contains 893,326 promoter methylation sequences, including 69,750 positive samples and 823,576 negative samples. Benchmark dataset 2, which focuses on non-small cell lung cancer (NSCLC), includes 1,335,158 promoter methylation sequences, with 170,484 positive samples and 1,164,674 negative samples. In both datasets, promoter fragments containing 5 mC sites were classified as positive samples, while those without 5 mC modifications were considered negative samples. The ratio of positive to negative samples is approximately 1:11.8 in benchmark dataset 1 and 1:6.8 in benchmark dataset 2. This class imbalance accurately reflects the natural distribution of 5 mC sites in promoters. To ensure a robust evaluation, both positive and negative samples were split into a training set and a test set at an 8:2 ratio. Then, using a stratified random sampling method, the training set was further divided, with 80% allocated for model training and 20% for validation. Details of the benchmark datasets are presented in Tables 1, 2.

**TABLE 1:** Detailed information on baseline dataset 1, SCLC.

Dataset	Positive sample	Negative sample
Training dataset	55,800	658,861
Testing dataset	13,950	164,715
Total	69,750	823,576

**TABLE 2:** Detailed information on baseline dataset 2, NSCLC.

Dataset	Positive sample	Negative sample
Training dataset	136,387	931,739
Testing dataset	34,097	232,935
Total	170,484	1,164,674

### 2.2 Feature coding methods

#### 2.2.1 One-Hot Encoding

One-Hot Encoding (Li et al., 2022) is a method that converts categorical variables into numerical representations. Each category is represented by a vector where only one element is 1, while all others are 0. The position of the 1 corresponds to the index of that category. Due to its simplicity and effectiveness, One-Hot Encoding is widely used in bioinformatics. For 5 mC site sequences, DNA is composed of four bases: adenine (A), cytosine (C), guanine (G), and thymine (T). These bases are encoded as follows: A (1,0,0,0), C (0,1,0,0), G (0,0,1,0), and T (0,0,0,1). As the sequences in this study have a fixed length of 41 base pairs (bp), each sequence is transformed into a 4 × 41 two-dimensional matrix after One-Hot encoding, providing a structured numerical representation for further analysis.

#### 2.2.2 NCP encoding

NCP Encoding (Luo et al., 2022) is a feature representation method based on three key chemical properties of nucleotides: ring structure, chemical functional groups, and hydrogen bond strength. Nucleotides are classified into purines (A, G) with two rings and pyrimidines (C, T) with one ring. Chemical functional groups also vary, with A and C containing amino groups (-NH_2_), while G and T contain keto groups (C=O). In terms of hydrogen bond strength, A-T pairs are held together by two hydrogen bonds, whereas G-C pairs form three hydrogen bonds, making them more stable. By encoding DNA sequences based on these properties, NCP Encoding provides a biologically meaningful representation that enhances feature extraction in computational models. To summarize, we use (Ai, Bi, Ci) to represent the first nucleotide in the sequence, where Ai, Bi, Ci are each of the above three chemical properties, expressed as follows:

$$A_{i}=\left\{\begin{array}{c} 1,p_{i}\in \left(A,G\right)\\ 0,p_{i}\in \left(C,T\right) \end{array}\right.B_{i}=\left\{\begin{array}{c} 1,p_{i}\in \left(A,C\right)\\ 0,p_{i}\in \left(G,T\right) \end{array}\right.C_{i}=\left\{\begin{array}{c} 1,p_{i}\in \left(A,T\right)\\ 0,p_{i}\in \left(C,G\right) \end{array}\right.$$

So, from above, we have adenine (A), cytosine (C), guanine (G), and thymine (T) as (1,1,1), (0,1,0), (1,0,0), and (0,0,1), respectively.

While the NCP feature values are a deterministic function of the nucleotide identity and could be derived from the One-hot encoding, we concatenate them explicitly to provide the model with a direct, interpretable, and biophysically meaningful prior on nucleotide chemical properties, aiming to improve learning efficiency and model performance.

### 2.3 Model construction

**FIGURE 1:**
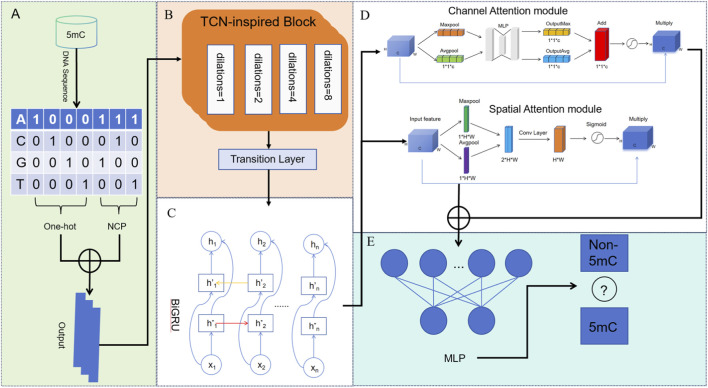
The architecture of the TCN-5mC model. **(A)** In the data preprocessing stage, input DNA sequences are encoded by combining One-hot and Nucleotide Chemical Property (NCP) encoding to create a comprehensive feature matrix. **(B)** The TCN-inspired block captures long-range dependencies, followed by a Transition Layer. **(C)** A Bidirectional Gated Recurrent Unit (BiGRU) to learn sequential contextual patterns. **(D)** Parallel attention modules allow concurrent enhancement of the feature representation. **(E)** The MLP network predicts whether a given DNA sequence is a 5 mC or non-5mC.

The overall architecture of our proposed TCN-5mC model is illustrated in Figure 1. The process begins with data preprocessing, where raw DNA sequences are transformed into a numerical format suitable for the deep learning model. As described in the above section, One-Hot encoding and NCP encoding are concatenated, creating a 7-dimensional feature matrix for each sequence of length 41 (7 x 41 matrix). Next, the encoded feature matrix enters the main network structure. This structure is a sequential pipeline of specialized neural network modules designed to learn complex patterns from the sequence data.

The first module is a TCN-inspired block. The TCN is composed of stacked layers of dilated causal convolutions. The architecture is highly effective for sequence data as it can capture long-range dependencies. The “causal” nature ensures that the prediction for a given position only depends on past information, which is natural for sequences. The “dilation” allows the network’s receptive field to grow exponentially with depth without a corresponding increase in computational cost, enabling it to efficiently model relationships between distant nucleotides in the DNA sequences. The block also incorporates residual connections, which help in training deeper networks by preventing the vanishing gradient problem (Bai et al., 2018).

The TCN-inspired block efficiently captures long-range dependencies through dilated convolutions, while BiGRU handles bidirectional sequence context. To prevent overfitting and enhance generalization, we introduced a transition layer between the TCN-inspired block output and BiGRU input. The transition layer consists of a convolutional layer with a kernel size of 1 and a pooling layer with a pooling window size of 4. After pooling, batch normalization is applied to improve the model’s generalization ability. The BiGRU module then processes the sequence. A GRU is a type of recurrent neural network (RNN) with gating mechanisms that control the flow of information, allowing it to selectively remember or forget information over long sequences (Chung, 2014). The “bidirectional” aspect means that the input sequence is processed in both forward and reverse directions by two separate GRU layers. The outputs from both directions are then concatenated. This allows the model to consider both the preceding and succeeding context for every nucleotide in the sequence, leading to a more robust and accurate understanding of its contextual significance (Tang, 2022).

The feature representation from the BiGRU is then refined by an improved Convolutional Block Attention Module (CBAM). This module is designed to highlight the most salient features while suppressing irrelevant ones. It consists of two parallel sub-modules: a Channel Attention Module and a Spatial Attention Module (Chen, 2017). The Channel Attention Module assigns different weights to each channel, emphasizing the most relevant information for the current task while suppressing less important features. This is typically achieved by capturing channel dependencies through global average pooling and max pooling, which generate channel descriptors. These descriptors are then processed through a shared fully connected layer and a sigmoid activation function to obtain the final channel attention weights. This method effectively enhances feature representation, improving the convolutional neural network’s ability to focus on important channel information. The Spatial Attention Module is a mechanism in convolutional neural networks that helps identify the most important regions in the input features. Its goal is to assign weights to different spatial locations, emphasizing regions that are more relevant to the task while suppressing less important areas.

Finally, the refined feature vector from the attention module is flattened and fed into a standard Multilayer Perceptron (MLP), which is a fully connected neural network (Wang et al., 2022). This MLP acts as the final classifier. It processes the high-level features and, using a softmax activation function in its output layer, calculates the probability of the input sequence containing a 5 mC site. The final output is a binary prediction: either “5 mC” or “Non-5mC”.

### 2.4 Performance evaluation

In this study, we used four common evaluation metrics to assess classifier performance: sensitivity (Sn), specificity (SP), accuracy (Acc), and Matthews correlation coefficient (MCC) (Sokolova and Lapalme, 2009). The specific calculation formulas are as follows:

$$\left\{\begin{array}{l} \begin{array}{l} \text{SP}=\frac{\text{TN}}{\text{TN}+\text{FP}}\\ \text{Sn}=\frac{\text{TP}}{\text{TP}+\text{FN}}\\ \text{Acc}=\frac{\text{TP}+\text{TN}}{\text{TP}+\text{TN}+\text{FP}+\text{FN}} \end{array}\\ \text{MCC}=\frac{\text{TP}*\text{TN}-\text{FP}*\text{FN}}{\sqrt{\left(\text{TP}+\text{FP}\right)*\left(\text{TP}+\text{FN}\right)*\left(\text{TN}+\text{FP}\right)*\left(\text{TN}+\text{FN}\right)}} \end{array}\right.$$

Here, TP (True Positive) refers to the number of positive samples correctly predicted as positive. FP (False Positive) represents negative samples incorrectly classified as positive. TN (True Negative) denotes negative samples correctly identified as negative, while FN (False Negative) refers to positive samples mistakenly classified as negative. Sensitivity (Sn) and specificity (SP) measure the proportion of correctly predicted positive and negative samples, respectively. Accuracy (Acc) represents the overall proportion of correct predictions, while the Matthews Correlation Coefficient (MCC) provides a balanced evaluation of model performance. Additionally, we use the ROC curve (Yang and Berdine, 2017) and compute the Area Under the ROC Curve (AUC) to assess the overall model performance. The AUC value ranges from 0 to 1, with values closer to 1 indicating better model performance.

### 2.5 Hyperparameter settings

In this study, all experiments were conducted under the same hyperparameter configuration to ensure reproducibility and fairness. The proposed deep learning model was trained using the Adam optimizer with an initial learning rate of 1 × 10^−3^, which was dynamically adjusted through a cosine decay schedule during training. The batch size was set to 64, and the maximum number of epochs was 100. To prevent overfitting, early stopping was applied with a patience of 10 epochs based on the validation loss. The focal loss function was employed to handle class imbalance, with a focusing parameter γ = 2 and a balance factor α = 0.25. The dropout rate was set to 0.5 for all dense and convolutional layers to improve generalization.

In the TCN, the number of filters was set to 128, with a kernel size of 3 and dilation rates of (Chen et al., 2016; Devi et al., 2021; Vanaja et al., 2009; Zhang et al., 2015). The BiGRU layer contained 128 hidden units in each direction, and the CBAM attention module was integrated after the BiGRU layer to enhance spatial and channel-wise feature extraction.

For the meta-learning integration, a two-layer MLP was trained with hidden units of [64, 32] and ReLU activation, using the Adam optimizer with a learning rate of 1 × 10^−4^.

## 3 Results and discussion

### 3.1 Compare various feature extraction techniques

In the proposed TCN-5mC network architecture, we compare the performance of three different feature encoding methods: One-hot encoding, NCP encoding, and a combination of One-hot and NCP encoding. One-hot encoding represents the nucleotide sequence of 5 mC sites as a 4 × 41 matrix, while NCP encoding transforms the sequence into a 3 × 41 matrix. The combined method, One-hot + NCP encoding, results in a 7 × 41 matrix, incorporating both encoding techniques to capture more detailed feature representations. The feature matrices generated by these three encoding methods were input into the TCN-5mc network. The experimental results on both the training and independent test sets are shown in Tables 3–6. The results indicate that combined One-hot encoding + NCP encoding performs best in terms of MCC. Since the data used in this experiment is imbalanced, the MCC metric is crucial for evaluation. The combined encoding method achieves a 5.3% and 1.8% higher MCC on the independent test set compared to the individual encoding methods, respectively. The combined encoding shows small but steady improvements in MCC. MCC measures how well a model works with imbalanced data. This means that giving the model both types of information helps it handle the imbalanced data problem better. Therefore, we choose to use the combined One-hot + NCP encoding for our model.

**TABLE 3:** Feature coding methods based on 5-fold cross-validation of the training dataset (benchmark dataset 1).

Encoding	Sn	SP	Acc	MCC	AUC
One-hot	0.6632 ± 0.0032	**0.9591** ± 0.0026	0.9358 ± 0.0042	0.5715 ± 0.0015	0.9644 ± 0.0011
NCP	0.6904 ± 0.0035	0.9568 ± 0.0064	**0.9360** ± 0.0028	0.5959 ± 0.0030	0.9645 ± 0.0014
One-hot + NCP	**0.7510** ± 0.0016	0.9512 ± 0.0045	0.9356 ± 0.0032	**0.6584** ± 0.0021	**0.9666** ± 0.0009

The highest value for each evaluation metric is highlighted in bold.

**TABLE 4:** Feature coding methods based on the independent test dataset (benchmark dataset 1).

Encoding	Sn	SP	Acc	MCC	AUC
One-hot	0.6477	**0.9603**	**0.9359**	0.5781	0.9644
NCP	0.7176	0.9544	**0.9359**	0.6061	0.9646
One-hot + NCP	**0.7396**	0.9522	0.9356	**0.6598**	**0.9666**

The highest value for each evaluation metric is highlighted in bold.

**TABLE 5:** Feature coding methods based on 5-fold cross-validation of the training dataset (benchmark dataset 2).

Encoding	Sn	SP	Acc	MCC	AUC
One-hot	0.8707 ± 0.0053	0.9771 ± 0.0049	0.9635 ± 0.0028	0.8381 ± 0.0019	0.9883 ± 0.0001
NCP	0.8567 ± 0.0037	**0.9797** ± 0.0035	0.9639 ± 0.0016	0.8380 ± 0.0035	0.9883 ± 0.0004
One-hot + NCP	**0.8748** ± 0.0036	0.9788 ± 0.0042	**0.9655** ± 0.0011	**0.8466** ± 0.0025	**0.9889** ± 0.0001

The highest value for each evaluation metric is highlighted in bold.

**TABLE 6:** Feature coding methods based on the independent test dataset (benchmark dataset 2).

Encoding	Sn	SP	Acc	MCC	AUC
One-hot	0.8680	0.9775	0.9635	0.8377	0.9883
NCP	0.8575	**0.9796**	0.9639	0.8380	0.9883
One-hot + NCP	**0.8744**	0.9787	**0.9655**	**0.8461**	**0.9889**

The highest value for each evaluation metric is highlighted in bold.

### 3.2 Ablation experiments of the model

We conducted ablation experiments on the network to determine the optimal combination of components. As there are two benchmark datasets in this study, we chose Dataset 2 for the ablation experiments. The results of the ablation experiments using five-fold cross-validation on Dataset 2 are shown in Table 7, and the results on the independent datasets are shown in Table 8. In the two tables, components are abbreviated as TCNiB (TCN-inspired Block), TL (Transition Layer), and BiGRU, with full descriptions provided in the legends. These tables evaluated four configurations: TCNiB + TL, TCNiB + BiGRU, TL + BiGRU, and TCNiB + TL + BiGRU, with the best results marked in bold.

**TABLE 7:** Ablation experiments based on 5-fold cross-validation of the training dataset.

The combination of components	Sn	SP	Acc	MCC	AUC
TCNiB + TL	0.8563 ± 0.0056	0.9791 ± 0.0059	0.9633 ± 0.0043	0.8359 ± 0.0065	0.9881 ± 0.0023
TCNiB + BiGRU	0.8700 ± 0.0085	0.9780 ± 0.0036	0.9632 ± 0.0072	0.8307 ± 0.0028	0.9881 ± 0.0023
TL + BiGRU	0.7523 ± 0.0053	0.9508 ± 0.0039	0.9353 ± 0.0097	0.6176 ± 0.0026	0.9646 ± 0.0015
TCNiB + TL + BiGRU	**0.8748** ± 0.0036	**0.9788** ± 0.0042	**0.9655** ± 0.0011	0**.8466** ± 0.0025	**0.9889** ± 0.0001

The highest value for each evaluation metric is highlighted in bold.

**TABLE 8:** Ablation experiments based on the independent test dataset.

The combination of components	Sn	SP	Acc	MCC	AUC
TCNiB + TL	0.8626	0.9782	0.9634	0.8367	0.9882
TCNiB + BiGRU	0.8663	0.9772	0.9631	0.8360	0.9875
TL + BiGRU	0.6994	0.9558	0.9358	0.5984	0.9646
TCNiB + TL + BiGRU	**0.8744**	**0.9787**	**0.9655**	**0.8461**	**0.9889**

The highest value for each evaluation metric is highlighted in bold.

The data in Tables 7, 8 show that the combination of all components yields the best performance. A comparison between the “TL + BiGRU” with “TCNiB + TL + BiGRU” models reveals that the latter outperforms the former, highlighting the importance of the TCN-inspired block in the overall architecture. When comparing “TCNiB + TL” with “TCNiB + BiGRU” models, the differences in all metrics are minimal. The “TCNiB + TL” model performs better in specificity, making it more likely to classify positive samples as negative, while the “TCNiB + BiGRU” model achieves better sensitivity, making it more likely to classify negative samples as positive. The network framework combining all methods extracts more advanced features than the other configurations. Therefore, we ultimately chose the combination of all components as the final network architecture for TCN-5mC.

### 3.3 Performance of TCN-5mC on the training dataset

**FIGURE 2:**
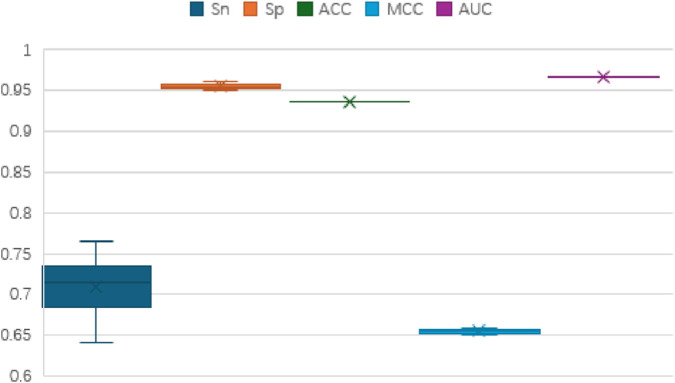
Boxplot analysis of the results of dataset 1.

**FIGURE 3:**
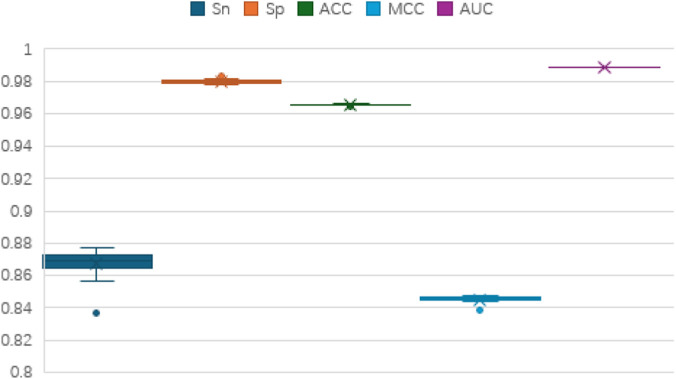
Boxplot analysis of the results of dataset 2.

To assess the performance of the TCN-5mC classifier, we conducted 5-fold cross-validation, repeating the process 20 times on the training datasets from two benchmark sets. The results, displayed in Figures 2, 3, show stable performance across the repetitions, suggesting the model has strong generalization ability. Figure 2 presents the outcomes for dataset 1, while Figure 3 presents the results for dataset 2. The model consistently performs well across key metrics, including Sn, Sp, ACC, MCC, and AUC.

### 3.4 Comparison with existing models

The performance of the TCN-5mC model was compared with that of existing models using five-fold cross-validation on Dataset 1 and Dataset 2, as shown in Tables 9, 10. Due to the imbalanced nature of the training data, the model tends to favor recognizing promoter fragments without 5 mC sites. As shown in Table 9, the DGA-5mC model achieves the highest Sn among all methods, but it also has the lowest SP, with a difference of 15.3%. In contrast, our model achieves a higher SP than other prediction methods, outperforming the latest BERT-5mC model by 1.4% and the highest BiLSTM-5mC model by 1.1%. In terms of Acc, our model exceeds the latest and highest-performing models by 0.4%. Given the imbalanced dataset, MCC is a crucial metric for evaluation. Our model achieves an MCC that is 0.5% higher and an AUC that is 0.1% higher than the best-performing model, demonstrating its superior performance. On Dataset 2, we compare our model with the BiLSTM-5mC model, as it is the only other model for which test results were available. The results in Table 10 show that our model outperforms BiLSTM-5mC in all metrics except Sn, with MCC being 4.4% higher. This highlights the strong generalization ability of our model.

**TABLE 9:** Comparison of the performance of different models on training dataset 1 using 5-fold cross-validation.

Predictor	Sn	SP	Acc	MCC	AUC
iPromoter-5mC	0.875	0.904	0.902	0.574	0.957
5 mC_Pred	0.899	0.920	0.918	0.626	0.962
BiLSTM-5mC	0.810	0.940	0.930	0.624	0.964
DGA-5mC	**0.904**	0.925	0.923	0.642	0.964
BERT-5mC	0.871	0.937	0.932	0.653	0.966
TCN-5mC	0.751 ± 0.0016	**0.951 ± 0.0045**	**0.936 ± 0.0032**	**0.658 ± 0.0021**	**0.967 ± 0.0009**

The highest value for each evaluation metric is highlighted in bold.

**TABLE 10:** Comparison of the performance of different models on training dataset 2 using 5-fold cross-validation.

Predictor	Sn	SP	Acc	MCC	AUC
iPromoter-5mC	**0.895**	0.920	0.931	0.786	0.981
BiLSTM-5mC	0.875	0.966	0.949	0.803	0.983
TCN-5mC	0.875 ± 0.0036	**0.979 ± 0.0042**	**0.966 ± 0.0011**	**0.847 ± 0.0025**	**0.989 ± 0.0001**

The highest value for each evaluation metric is highlighted in bold.

Furthermore, we compared our model with existing models on the independent test datasets, as shown in Tables 11, 12. On the independent test dataset 1 (Table 11), our model achieves the highest performance in all metrics except Sn. Specifically, our model’s SP, Acc, MCC, and AUC are 1.4%, 0.3%, 0.4%, and 0.1% higher than the best-performing existing methods, respectively, demonstrating the superiority of our model. On the independent test dataset 2 (Table 12), our model continues to perform exceptionally well. Compared to the BiLSTM-5mC model, our SP, Acc, MCC, and AUC are higher by 1.4%, 1.6%, 3.9%, and 0.06%, respectively. Overall, the TCN-5mC model achieves outstanding performance on both the training and independent test datasets, outperforming other existing predictors. These results indicate that TCN-5mC has a stronger generalization ability and improved prediction accuracy in identifying potential 5 mC sites.

**TABLE 11:** Comparison of the performance of different models on the independent test dataset 1.

Predictor	Sn	SP	Acc	MCC	AUC
iPromoter-5mC	0.878	0.904	0.902	0.577	0.957
5 mC_Pred	0.895	0.920	0.918	0.625	0.962
BiLSTM-5mC	0.866	0.937	0.930	0.638	0.964
DGA-5mC	**0.902**	0.927	0.925	0.646	0.964
BERT-5mC	0.872	0.938	0.933	0.656	0.966
TCN-5mC	0.740	**0.952**	**0.936**	**0.660**	**0.967**

The highest value for each evaluation metric is highlighted in bold.

**TABLE 12:** Comparison of the performance of different models on the independent test dataset 2.

Predictor	Sn	SP	Acc	MCC	AUC
iPromoter-5mC	**0.894**	0.921	0.931	0.787	0.981
BiLSTM-5mC	0.884	0.965	0.950	0.807	0.983
TCN-5mC	0.874	**0.979**	**0.966**	**0.846**	**0.989**

The highest value for each evaluation metric is highlighted in bold.

## 4 Conclusion

This study introduced TCN-5mC, a novel deep learning framework that significantly improves the prediction of 5 mC methylation sites within promoter regions. By synergistically combining a TCN-inspired block with a Bidirectional Gated Recurrent Unit (BiGRU), our model effectively captures both long-range sequence dependencies and local contextual features. Furthermore, the integration of a Convolutional Block Attention Module (CBAM) refines the learned representations by adaptively highlighting the most salient spatial and channel-wise features, thereby enhancing the model’s discriminative power. A key strength of TCN-5mC is its exceptional performance on highly imbalanced, real-world datasets from human SCLC and NSCLC cell lines, where it achieves high accuracy without requiring artificial sample balancing. This demonstrates the model’s robustness and strong generalization capabilities in a realistic application scenario.

The success of TCN-5mC has important implications for the field of computational epigenetics. Accurate, high-throughput prediction of 5 mC sites can accelerate research into the complex mechanisms of gene regulation in cancer and facilitate the discovery of novel epigenetic biomarkers for diagnostic and prognostic purposes.

Future work should focus on extending the application of TCN-5mC to a broader range of cancer types and other methylation-related diseases. Furthermore, integrating multi-omics data, such as gene expression and chromatin accessibility, could further enhance the model’s predictive power. Exploring model interpretability to extract biologically meaningful patterns from the learned representations also presents a promising avenue for subsequent research.

In conclusion, TCN-5mC represents a powerful and reliable computational tool that advances our ability to decode the epigenetic landscape, offering significant potential for both basic and translational research.

While the TCN-5mC model demonstrates superior performance on lung cancer promoter datasets, we acknowledge a limitation of the current study: the evaluation was conducted within the specific context of lung cancer. This focused approach was necessary to ensure a direct and methodologically fair comparison with existing state-of-the-art predictors, which were also developed and benchmarked exclusively on lung cancer data. Consequently, the generalizability of TCN-5mC to other tissue types or across different species remains to be fully established. Future work will be essential to validate and, if necessary, adapt the model across diverse datasets encompassing various cancers and biological contexts. Such cross-tissue and cross-species validation will be a critical step in assessing the broader applicability of the model and in translating computational predictions into more universal epigenetic insights.

## Data Availability

Publicly available datasets were analyzed in this study. This data can be found here: https://github.com/lcw1103/5mc.
